# Evidence of a Down Syndrome Keratopathy: A Three-Dimensional (3-D) Morphogeometric and Volumetric Analysis

**DOI:** 10.3390/jpm11020082

**Published:** 2021-01-30

**Authors:** Ibrahim Toprak, Francisco Cavas, Alfredo Vega, José S. Velázquez, Jorge L. Alio del Barrio, Jorge L. Alio

**Affiliations:** 1 Department of Research and Development, VISSUM, 03016 Alicante, Spain; ibrahimt@doctor.com (I.T.); alfredovega@vissum.com (A.V.); jorge_alio@hotmail.com (J.L.A.d.B.); 2 Department of Ophthalmology, Faculty of Medicine, Pamukkale University, Denizli 20160, Turkey; 3 Department of Structures, Construction and Graphical Expression, Technical University of Cartagena, 30202 Cartagena, Spain; jose.velazquez@upct.es; 4 Cornea, Cataract and Refractive Surgery Department, VISSUM, 03016 Alicante, Spain; jlalio@vissum.com; 5 Division of Ophthalmology, Department of Pathology and Surgery, Faculty of Medicine, Miguel Hernández University, 03016 Alicante, Spain

**Keywords:** corneal ectasia, personalized corneal model, corneal volume, down syndrome, irregular astigmatism, keratoconus, keratopathy, visual optics

## Abstract

The aim of this study was to investigate whether a different and abnormal corneal profile is present in Down syndrome (DS) by personalized three-dimensional (3D) modelling. This single-centre cross-sectional study included 43 patients with DS (43 eyes) and 58 age-sex-matched control subjects (58 eyes) with normal karyotype and topography. Refraction, central corneal thickness (CCT), aberrations (high-order, coma and spherical), asphericity and morphogeometric/volumetric parameters based on a 3D corneal model that was generated from raw topographical data were evaluated. Deviation of anterior/posterior apex (D_apexant_/D_apexpost_) and thinnest point (D_mctant_/D_mctpost_) from corneal vertex, anterior/posterior surface area (A_ant_/A_post_), sagittal area passing through the anterior/posterior apex (A_apexant_/A_apexpost_) and thinnest point (A_mctpost_), total corneal volume (V_total_) and volumetric progression for each 0.05 mm step of the radius value centred to the thinnest point (VOL_MCT_) and anterior/posterior apex (VOL_AAP_/VOL_PAP_) comprised the morphogeometric/volumetric parameters. In the DS group, 58.1% of the eyes presented abnormal topography. High-order and coma aberrations, asphericity, D_apexant_, A_ant_, A_post_ and A_apexant_ were significantly higher, whereas CCT, A_apexpost_, A_mctpost_, V_total_, VOL_AAP_, VOL_PAP_ and VOL_MCT_ were lower in the DS group than in the control group (*p* < 0.05). D_apexpost_ did not differ between the groups (*p* > 0.05). This study demonstrates that corneas of the subjects with DS are different and more aberrated than those of normal age- and sex-matched non-DS controls. Anterior corneal apex appears to be displaced in DS even with normal topography, while posterior apex seems stable although topography is abnormal. These findings may help to modify our approach in the diagnosis of keratopathy in subjects with DS.

## 1. Introduction

Down syndrome (DS) is the most common genetic disorder caused by the presence of an extra copy of chromosome 21, and it is associated with cognitive impairment and multisystemic malformations [1,2]. Up-slanting palpebral fissures, epiblepharon, epicanthal folds, nystagmus, strabismus, accommodative insufficiency, high refractive errors, keratoconus (KC), presenile cataract, glaucoma and retinovascular anomalies are the common ophthalmic disorders seen in patients with DS [1,2,3].

KC occurs in about 1–15% of patients with DS [4]. However, a recent study by Alio et al. reported that 71.3% of patients with DS present corneal topographical abnormalities compatible with KC [5].

Initially, the strong link between DS and KC was ascribed to the genes on chromosome 21 affecting collagen metabolism, oxidative stress and degradation; however, this hypothesis was not confirmed, and several studies concluded that genetic heterogenicity that interacts with environmental factors were responsible for KC development [6,7,8,9].

Several studies have suggested that patients with DS had steeper, thinner and more irregular corneas than those from non-DS controls, even though these findings did not fulfil the criteria for KC diagnosis [5,10,11,12,13,14]. However, Alio and co-workers [5] were the first to describe the high frequency of abnormal topographical findings in patients with DS.

Previous studies were mainly based on corneal topographic or pachymetric assessments. However, the three-dimensional (3D) cornea characteristics in DS have not yet been presented, and might provide critical information to understand the corneal structural properties of DS corneas.

In recent years, Cavas et al. [15,16,17] introduced an innovative 3D personalized virtual model that allows point to point, areal and volumetric analysis of the cornea. This customized 3D model presumes that deteriorations in morphogeometric and volumetric variables could originate from abnormalities in the corneal collagen matrix organization, and this theory was confirmed in the diagnosis of subclinical and clinical KC in previous studies [15,16,17,18].

This study was designed to investigate whether DS cases have a different and abnormal corneal profile than that of age- and sex-matched non-DS subjects with normal topography using a 3D model.

## 2. Materials and Methods

The tenets of the Declaration of Helsinki on the use of human subjects in research were followed, and institutional ethics committee approval was obtained for this research. The study data was provided from VISSUM Innovation (Cornea, Cataract and Refractive Surgery Unit, Alicante, Spain) affiliated by Miguel Hernandez University (Elche, Spain) and the Keratoconus IBERIA database.

### 2.1. Study Design and Population

This cross-sectional study included 43 consecutive DS patients (43 eyes) with genotypic confirmation that were provided from local DS institutions, and 58 consecutive non-DS refractive surgery candidates with normal topography (Sirius System^®^, CSO, Firenze, Italy) that did not develop corneal ectasia after 2 years post laser refractive surgery as control subjects (58 eyes). Preoperative topographic data of the non-DS subjects were used for the analysis.

### 2.2. Inclusion and Exclusion Criteria

The DS and control groups were matched based on age and gender in order to allow comparison. Subjects with good cooperation in ocular examinations and corneal topography measurements (DS group in particular) were included into the study. In bilateral cases, one eye was selected randomly using the Statistical Package for Social Sciences (SPSS) version 24 software (IBM SPSS Statistics Inc., Chicago, IL, USA). History of corneal surgery, hydrops, scarring, infection, other corneal thinning disorders, poor cooperation in topography measurements and low topographic test quality (quality score < 90%) were defined as the exclusion criteria.

### 2.3. Examination and Measurements

All subjects underwent a complete ophthalmological examination that included corrected distance visual acuity (CDVA, Snellen) assessment (when possible), slit-lamp biomicroscopy, tonometry, dilated fundus examination, retinoscopy and anterior segment tomography (Sirius System^®^, CSO, Firenze, Italy).

Patients were asked not to wear their contact lenses for 2 and 4 weeks prior to the measurements for the soft and hard contact lenses (if any), respectively.

A single experienced optometrist performed the topography measurements with proper head position of the patient. Three measurements were taken for each eye, and the one with the best image acquisition quality (coverage and centration scores over 90% with a green coloured checkmark) were used for statistical analysis.

### 2.4. Clinical and Topographical Classification

Clinical and topographical assessment was performed by three experienced cornea specialists (J.A., A.V. and J.A.B.). The Sirius System^®^ topographic classifier outputs (“Normal”, “KC suspect”, “KC compatible” and “Abnormal or treated”), which were confirmed by three observers with consensus, were used for topographical classification in order to prevent bias.

KC diagnosis was verified using the following criteria: presence of retinoscopic and biomicroscopic signs of KC such as scissoring, Vogt’s striae, Fleischer’s ring, Munson’s sign and Rizzuti’s phenomenon, any typical pattern for KC on axial/tangential curvature map (round, oval, superior steep, inferior steep, irregular, inferior-steep asymmetric bowtie, superior-steep asymmetric bowtie) and symmetric or asymmetric bowtie with skewed radial axes (SRAX) >21 degrees and concurrent central/paracentral or inferior focal steepening (anterior and/or posterior) and/or corneal thinning, and inferior–superior (I–S) keratometric difference >1.5D.

Additionally, considering the Sirius System^®^ classifier results, borderline topographical changes that did not fulfil the above-mentioned KC criteria were accepted as “KC suspect”. Uncategorized topographical findings that did not fit any kind of corneal ectasia pattern were classified as “Abnormal or treated”. Normal topographical maps without any above-mentioned abnormalities were classified as “Normal”.

### 2.5. Aim of the Study and Main Outcome Measures

The current study aimed to investigate whether subjects with DS had a different and abnormal corneal profile when compared to the non-DS controls. A further analysis was also performed based on topographic classification. The DS group was divided into two subgroups as “DS with normal topography” and “DS with abnormal topography” (including KC compatible, KC suspect and Abnormal or treated eyes) based on the topographical classification. Age, gender, sphere, cylinder, spherical equivalent, central corneal thickness (CCT), corneal aberrations (total, high-order, coma and spherical), 8-mm Q (asphericity) value and 3D morphogeometric and volumetric parameters were compared between the control and DS groups, and among the control and DS subgroups.

### 2.6. 3D Corneal Modelling, Morphogeometric and Volumetric Parameters

**Figure 1:**
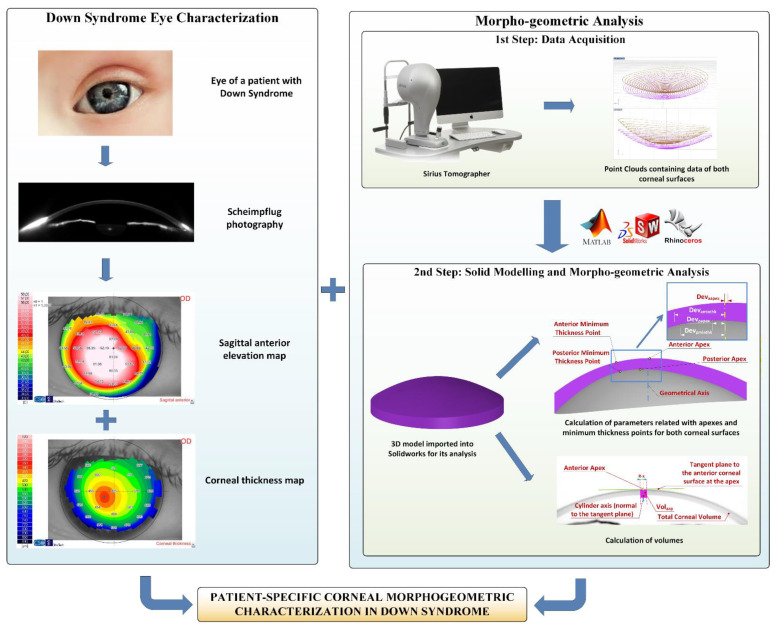
The steps comprising the morphogeometric characterization procedure in the Down syndrome group.

The morphogeometric characterization procedure applied in this research work was built upon the following steps (Figure 1).

*First step—Data acquisition:* The corneal tomography device used for this study permits the extraction of a cloud of points representative of the corneal surfaces by using its artificial vision algorithm. These point clouds are a discrete set of data in an M × N matrix format, so we relied upon a self-developed algorithm, which was later automated using Matlab^®^ R2018b (Mathworks, Natick, MA, USA) software, to covert the data of the coordinates in each topography file into Cartesian format, before their exportation to a CSV file [16,17]. Each row corresponds to a circle drawn over the corneal map, and each column relates to a semi-meridian (for each radius, 256 points were taken). Row samples were acquired following a circular path of radius i*0.2 mm, with “i” being the row number; and column samples were acquired following a semi-meridian in the path of j*360/256u, with “j” being the column number. The final result was a [i, j] matrix, in which each Z value defines the point P (i*0.2, j*360/256u) in polar coordinates. With this configuration, a point cloud was generated for the zone that started in the cornea’s geometric centre (r = 0 mm) and reached the mid-peripheral area (r = 4 mm). This area of study typically includes most of the biometrical information of the cornea, not only for healthy eyes, but also for diseased ones [16,17,19,20,21].

*Second step—Solid modelling and morphogeometric analysis:* The point cloud that reflects the corneal biometry was later incorporated into Rhinoceros^®^ V5.0 (MCNeel & Associates, Seattle, WA, USA) surface reconstruction software. To enhance the fitting between the generated surface and the point cloud, we relied upon Rhinoceros^®^ “patch” surface functionality, which minimizes the spatial distancing between the generated surface and the three-dimensional cloud. Finally, the surfaces obtained in previous steps were then exported to SolidWorks^®^ V2018 (Dassault Systèmes, Vélizy-Villacoublay, France) solid modelling software. By means of this software, the 3D custom model representing the corneal biometry was generated, and the following morphogeometric parameters could be calculated: anterior/posterior apex deviation from the geometrical axis (D_apexant_/D_apexpost_); anterior/posterior minimum thickness points deviation from the geometrical axis (D_mctant_/D_mctpost_); anterior corneal surface (ACS) area (A_ant_); posterior corneal surface (PCS) area (A_post_); total corneal surface area (A_tot_); area of the cornea within the sagittal plane passing through the Z axis and the anterior apex/posterior apex/posterior minimum thickness point (A_apexant_/A_apexpost_/A_mctpost_); centre of mass coordinates X,Y and Z (C_x_, C_y_, C_z_); total corneal volume (V_total_); volume between the solid corneal model and a cylinder with radius “x” (from 0.1 to 1.5 mm) and axis defined by the perpendicular line to the plane that is tangent to the anterior/posterior corneal surface at the apex (VOL_AAP_/VOL_PAP_); and volume between the solid corneal model and a cylinder with radius “x” (from 0.1 to 1.5 mm) and axis defined by the line joining points of minimum corneal thickness of both ACS and PCS (VOL_MCT_). All these indices have already been exhaustively described and used in precedent research from our group [16,17,19,20,21,22].

**Figure 2:**
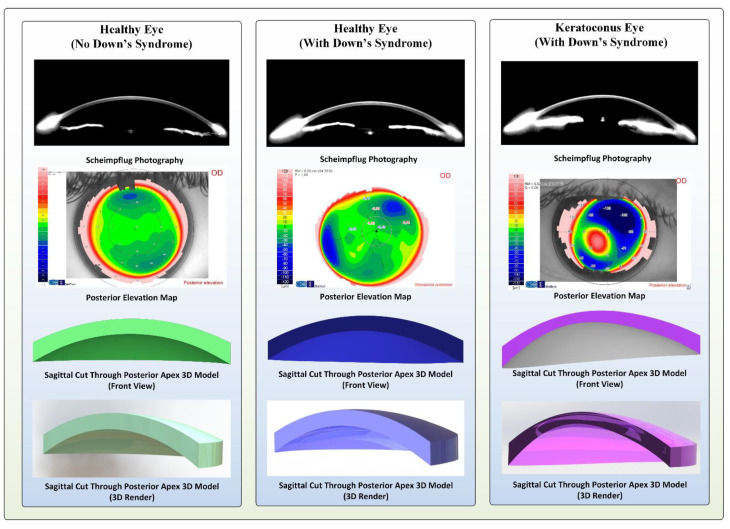
Topographical and three-dimensional (3D model) images of the cornea for sample cases in the control, Down syndrome (DS) with normal topography and DS with abnormal topography groups.

In conclusion, for each patient a topographic 3-D modelling analysis was performed (Figure 2).

### 2.7. Sample Size Calculation

Assuming an effect size (d) of 0.75, the minimum sample size was found to be 40 eyes per group (control and DS) at 95% power and 95% confidence level (G*Power version 3.1.9.6 computer software, Universität Düsseldorf, Düsseldorf, Germany).

### 2.8. Statistical Analysis

Statistical analysis was performed using SPSS version 24 (IBM SPSS Statistics Inc., Chicago, IL, USA) software. Qualitative data (gender) was compared between the groups using the chi square test. Normal distribution of the variables was tested with Kolmogorov–Smirnov test. Quantitative variables (age, refractive, topographic, pachymetric, aberrometric, morphogeometric and volumetric data) were expressed as mean ± standard deviation (SD). An independent samples *t*-test (Student’s *t*-test) was performed to compare quantitative data between the control and DS groups when parametric test assumptions were met. Otherwise, Mann-Whitney U test was used.

Kruskal-Wallis test with post-hoc correction was performed to compare quantitative variables among the control, DS with normal topography and DS with abnormal topography groups.

A *p*-value < 0.05 was accepted as statistically significant at 95% confidence interval (CI). After post-hoc correction, a *p*-value < 0.0166 indicated statistical significance.

## 3. Results

The DS (*n* = 43) and control (*n* = 58) groups were matched based on age and gender distribution (*p* > 0.05, Table 1), and were not showing statistically significant difference regarding sphere and spherical equivalent values (*p* > 0.05, Table 1). In the DS group, 18/43 (41.9%) eyes had normal topography and 25/43 (58.1%) eyes represented abnormal topography, which were classified as “KC compatible” (*n* = 7), “KC suspect” (*n* = 9) and “abnormal or treated” (*n* = 9).

### 3.1. Control vs. DS Group (Overall) Comparisons

#### 3.1.1. Refractive, Pachymetric and Aberrometric Parameters

Cylinder, root-mean-square (RMS) values for high-order and coma aberrations and 8-mm Q value were significantly higher (absolute values) in the DS group when compared to those of the control group (*p* < 0.05, Table 1). However, spherical aberration RMS value and CCT were found to be lower in the DS group than in the control group (*p* > 0.05, Table 2).

#### 3.1.2. 3D Morphogeometric and Volumetric Parameters

The DS group had significantly higher D_apexant_, D_mctant_, D_mctpost_, A_ant_, A_post_, A_apexant_ and C_z_ values compared to those of the control group (*p* < 0.05, Table 2). However, A_apexpost_, A_mctpost_, C_x_, and C_y_ values were lower in the DS group than in the control group (*p* < 0.05).

**Figure 3:**
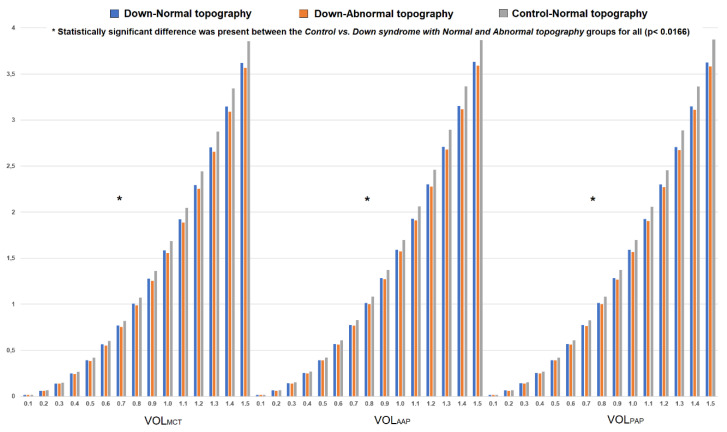
Change in corneal volume for each 0.05 mm step of the radius value (up to 1.5 mm in diameter) centred to the thinnest point (VOL_MCT_) and corneal apex (VOL_AAP_ and VOL_PAP_) in the control, Down syndrome (DS) with normal topography and DS with abnormal topography groups.

The DS group had lower V_total_ (23.60 ± 1.82 vs. 25.43 ± 1.62 mm^3^, *p* < 0.0001, Student’s *t*-test), VOL_MCT_, VOL_AAP_ and VOL_PAP_ for each 0.05 mm step of the radius value within 0.1 to 1.5 mm diameter when compared to those of the control group (*p* < 0.0001, Student’s *t*-test) (Figure 3). Furthermore, volumetric increase between 0.1 mm and 1.5 mm of the diameter values centred to the thinnest point (MCT, 3.57 ± 0.21 vs. 3.83 ± 0.26 mm^3^), anterior (AAP, 3.59 ± 0.25 vs. 3.86 ± 0.26 mm^3^) and posterior apex (PAP, 3.58 ± 0.25 vs. 3.85 ± 0.26 mm^3^) (*p* < 0.0001 for all, Student’s *t*-test) were also significantly decreased in the DS group.

#### 3.1.3. Correlations between Corneal Aberrations and Morphogeometric Parameters

In the control group, there were no statistically significant correlations between corneal aberrations and 3D morphogeometric/volumetric parameters. However, in the DS group, high-order aberrations (HOAs) (r = 0.542 *p* < 0.0001, r = 0.416 *p* = 0.005 and r = 0.389 *p* = 0.010) and coma RMS (r = 0.522 *p* < 0.0001, r = 0.500 *p* = 0.001 and r = 0.438 *p* = 0.003) were significantly correlated with D_apexant_, D_apexpost_ and C_z_, respectively. On the other hand, there were significant negative relations between spherical aberration vs. V_total_ (r = −0.354 *p* = 0.020), A_apexant_ (r = −0.343 *p* = 0.024), A_apexpost_ (r = −0.338 *p* = 0.027), A_mctpost_ (r = −0.329 *p* = 0.031), D_apexant_ (r = −0.452 *p* = 0.002) and C_z_ (r = −0.436 *p* = 0.003).

### 3.2. Control, DS with Normal Topography and DS with Abnormal Topography Group Comparisons

The DS with normal topography group was younger than the DS with abnormal topography and control groups (*p* < 0.05, Table 1).

#### 3.2.1. Refractive, Pachymetric and Aberrometric Parameters

Cylinder (absolute value) and coma RMS values were higher, and spherical aberration RMS was lower in the DS with abnormal topography group when compared to the control group (*p* < 0.05, Table 1). Both DS with normal and abnormal topography groups had lower 8-mm Q value (more negative) and CCT when compared to the control group (*p* < 0.05, Table 1). The HOA RMS value showed a significant trending increase among the control, DS with normal and abnormal topography groups, in that order (*p* < 0.05, Table 1). The remaining parameters were similar among the three groups (*p* > 0.05, Table 1).

#### 3.2.2. 3D Morphogeometric and Volumetric Parameters

D_apexant_ was significantly higher in the DS with normal and abnormal topography groups than in the control group (*p* < 0.05, Table 2), whereas D_apexpost_ was similar among the three groups (*p* > 0.05, Table 2). Moreover, the DS with abnormal topography group had higher D_mctant_ and D_mctpost_ values than in the control group. A_ant_, A_post_ and A_apexant_ were lower; and A_apexpost_, A_mctpost_ and C_x_ were higher in the control group when compared to the DS with normal and abnormal topography groups (*p* < 0.05, Table 2). The control group had lower C_z_ than the DS with normal topography group (*p* < 0.05, Table 2).

Regarding volumetric parameters, both DS with normal and abnormal topography groups had decreased V_total_ (23.69 ± 1.68 and 23.54 ± 1.95 vs. 25.43 ± 1.62 mm^3^, respectively), VOL_MCT_, VOL_AAP_ and VOL_PAP_ for each 0.05 mm step of the radius value (between 0.1 to 1.5 mm diameter) compared to the control group (*p* < 0.0166, Kruskal–Wallis test with post-hoc correction). Moreover, volumetric progression from 0.1 mm to 1.5 mm in diameter centred to the thinnest point (MCT), anterior (AAP) and posterior (PAP) apex were also significantly decreased in the DS with normal topography (MCT = 3.60 ± 0.18 mm^3^, AAP = 3.61 ± 0.21 and PAP = 3.60 ± 0.21 mm^3^) and DS with abnormal topography (MCT = 3.54 ± 0.24 mm^3^, AAP = 3.57 ± 0.27 and PAP = 3.56 ± 0.28 mm^3^) groups than in the controls (MCT = 3.83 ± 0.26 mm^3^, AAP = 3.86 ± 0.26 and PAP = 3.85 ± 0.26 mm^3^) (*p* < 0.010 for all, Kruskal–Wallis test with post-hoc correction).

Figure 3 represents volumetric distribution graphs in the control and DS groups (with subgroups).

## 4. Discussion

The current study demonstrates conclusively that the DS corneas were different and more irregular than matched controls of non-DS cases. Furthermore, this is the first study reporting the characterization of the cornea in patients with DS using novel morphogeometric and volumetric parameters derived from a 3D virtual model. Patients with DS had thinner and more aberrated corneas (high-order and coma) when compared to non-DS controls. These findings agree with recently published topography-based studies [5,11,12,13,14,23,24]. The 3D morphogeometric analysis in our study revealed that anterior corneal apex (D_apexant_) and the thinnest point (both anterior and posterior surfaces; D_mctant_ and D_mctpost_) showed significant deviation from corneal vertex in subjects with DS compared to controls. Pairwise comparisons demonstrated that the amount of anterior apex deviation was significantly higher in the DS with normal and abnormal topography groups than in the control group, while thinnest point deviation differed only between the DS with abnormal topography and control groups. This might indicate corneal pathological changes in DS manifest as thinnest-point deviation (as it does in the non-DS population with KC). On the other hand, posterior apex displacement (D_apexpost_) did not differ among the control, DS with normal and abnormal topography groups. In other words, for a normal (or abnormal) cornea in DS, anterior corneal apex seems to be displaced from vertex when compared to the non-DS subjects with normal cornea. Conversely, for an abnormal cornea in DS (classified as “KC compatible”, “KC suspect” and “abnormal or treated”), posterior apex deviation appears to be similar to that in non-DS population with normal topography. These findings conflict with our previous knowledge on corneal dynamics in KC in the non-DS population, which manifest as significant anterior and posterior apex displacement. Recent studies by our research group also demonstrated significant deviation in corneal apex (both anterior and posterior) and thinnest point in different stages of KC [15,16,17,18]. These findings might be important to point out differences in corneal structure and biomechanics between DS and non-DS subjects. On the other hand, anterior and posterior apex deviation appears to be correlated (positively) with high-order and coma aberrations in the DS group.

Anterior and posterior surface area (A_ant_ and A_post_), as well anterior sagittal area at corneal apex (A_apexant_) were increased in the DS groups (normal and abnormal topography subgroups). These findings might indicate the presence of anterior and posterior corneal surface irregularities such as focal elevations enlarging the surface area [18]. However, at the posterior corneal apex (A_apexpost_) and thinnest location (A_mctpost_), sagittal area was decreased in patients with DS, which could be due to a lowering effect of the corneal thinning at these locations, since corneal thickness is one of the variables in sagittal area calculation [15,16,17,18]. That is to say, the posterior apical point also appears to be located where the cornea is thinner, unlike the anterior apex in both DS subgroups. On the other hand, sagittal area values at apex and the thinnest point, as well as anterior apex displacement were negatively (at moderate strength) correlated with spherical aberration.

Centre of mass coordinates (x, y, z) of the cornea were significantly different in patients with DS from those of the non-DS controls, where the z value was higher and x, y values were lower in the DS group. This behaviour (increase of z coordinate along with a reduction of x and y coordinates) has already been observed for the most advanced stages of KC in non-DS subjects [19,20], and can be explained by the fact that the deformation suffered by both corneal surfaces generates a cone-like bulge that makes the centre of mass displace in the direction of the z coordinate. The higher this deformation, the most likely it is that apices and MCT coordinates tend to “realign” with the origin of coordinates, and therefore x and y diminish.

The patients with DS had significantly decreased total corneal volume (V_total_). Furthermore, corneal volume for each 0.05 mm step of the radius value (up to 1.5 mm in diameter) emerging from the thinnest point (VOL_MCT_) and corneal apex (VOL_AAP_ and VOL_PAP_) were also reduced in the DS group compared to in the controls. The thinner cornea in the DS group possibly led to diminished corneal volume. Several clinical studies have published similar results. For instance, Asgari et al. [12] reported reduced corneal volume in patients with DS regardless of the presence of KC. Hashemi et al. [23] agreed that DS patients without KC had homogenously distributed thin corneas with decreased volume. This study uses three points as the centre of the volumetric distribution, and change in corneal volume between 0.1 mm and 1.5 mm in diameter was the lowest in the DS with abnormal topography group, then in the DS with normal topography and control groups, in that order. The statistically significant difference was seen between the control and both DS subgroups. It seems that patients with DS had decreased corneal volume and volumetric progression even though the topography was normal. This finding might be associated with abnormal collagen structure, metabolism and organization attributed to DS, whereas there is no clear evidence confirming this idea [6,7,9,25]. On the other hand, a further comparative analysis between keratoconic corneas of DS and non-DS populations might add value for a better understanding of corneal structure and behaviour in DS.

From a practical perspective, the thinner and steeper nature of the cornea in patients with DS may affect the decision-making process of the clinician in diagnosing early KC in these cases, since normal characteristics of the cornea in DS might interfere with the diagnostic criteria set for the non-DS population [13,26]. For instance, a clinical study by Marsack et al. [26] showed that if inferior–superior keratometric difference (I–S) and the KISA% index were used for KC detection, 20.8% and 11.8% of the eyes (respectively) in subjects with DS exceeded the diagnostic threshold. Furthermore, if steep keratometry was used to grade KC severity based on the Collaborative Longitudinal Evaluation of Keratoconus (CLEK) criteria in DS patients that met either I–S or KISA% criteria, 89.3% of the eyes represented moderate KC (steep keratometry between 45 and 52 dioptres). The authors also strongly emphasized that their findings did not mean that all these cases with DS had KC, but the cornea in subjects with DS exhibited clinical features very similar to KC.

## 5. Conclusions

The results of the current study strongly suggest that patients with DS have a different and more aberrated cornea profile with decreased volume, even when topography is considered normal. Based on the morphogeometric analysis, the anterior apex tended to deviate from corneal vertex in DS patients with normal or abnormal topography, as seen in KC. However, in DS, regardless of topography, posterior corneal apex displacement seemed not to differ from the non-DS population with normal topography, in contrast to what has previously been seen in non-DS KC. Therefore, the corneal characteristics presented in this study might be valuable and pioneering for re-defining perspectives in the diagnosis of keratopathy in DS, where early detection and prompt treatment are critical due to additional systemic and intellectual problems. On the other hand, in future research it would be interesting to compare the morphogeometric characteristics of the keratoconic corneas between the DS and non-DS populations.

## Figures and Tables

**Table 1:** Comparison of the control and Down syndrome groups regarding age, gender, refractive, aberrometric and pachymetric data.

Variables (Mean ± SD)	Control Group Normal Topography (*n* = 58)	Down Syndrome Normal Topography (*n* = 18)	Down Syndrome Abnormal Topography (*n* = 25)	Down Syndrome Group Total (*n* = 43)	3-Group Comparison * p	Pairwise Comparisons ** p	Down vs. Control Group Comparison *** p
Age (years)	**26.9 ± 10.1**	**17.7 ± 8.1**	**29.0 ± 11.0**	24.3 ± 11.3	**0.002**	**a, c (0.004, 0.002)**	0.224 (S)
Gender (F/M)	30/28	11/7	13/12	24/19	0.773 (chi square)	-	0.693 (C)
Sphere (dioptres)	−0.74 ± 3.80	0.18 ± 5.31	0.10 ± 4.03	0.13 ± 4.56	0.095	-	0.085 (M)
Cylinder (dioptres)	**−0.58 ± 0.62**	−1.23 ± 0.84	**−2.03 ± 1.04**	**−1.69 ± 1.03**	**<0.0001**	**b (<0.0001)**	**<0.0001** (M)
Spherical equivalent (dioptres)	−1.03 ± 3.78	−0.43 ± 5.25	−0.91 ± 4.00	−0.70 ± 4.52	0.199	-	0.120 (M)
High-order aberrations (HOAs) RMS (μm)	**0.40 ± 0.10**	**0.63 ± 0.41**	**1.64 ± 1.24**	**1.22 ± 1.10**	**<0.0001**	**a, b, c (0.011, <0.0001, 0.006)**	**<0.0001** (M)
Coma RMS (μm)	**0.26 ± 0.10**	0.39 ± 0.16	**0.98 ± 1.17**	**0.73 ± 0.94**	**<0.0001**	**b (<0.0001)**	**<0.0001** (M)
Spherical RMS (μm)	**0.21 ± 0.05**	0.15 ± 0.19	**0.002 ± 0.67**	**0.06 ± 0.52**	**0.004**	**b (0.004)**	**0.001** (M)
8-mm Q value	**−0.21 ± 0.18**	**−0.42 ± 0.10**	**−0.53 ± 0.30**	**−0.49 ± 0.24**	**<0.0001**	**a, b (<0.0001 both)**	**<0.0001** (M)
Central corneal thickness (μm)	**544.2 ± 32.5**	**502.5 ± 24.8**	**491.5 ± 33.4**	**500.8 ± 29.5**	**<0.0001**	**a, b (0.001, <0.0001)**	**<0.0001** (S)

F/M: female/male; KC: keratoconus; RMS: root mean square; SD: standard deviation. *p* < 0.05 and bold values indicate statistical significance. * Comparison of the control group (non-Down subjects with normal topography), Down syndrome with normal topography and Down syndrome with abnormal topography subgroups (Kruskal-Wallis test, *p* < 0.05 indicates statistically significant difference). ** Pairwise comparisons among the three groups (*p* < 0.0166 indicates statistically significant difference after post-hoc corrections); “a” indicates a statistically significant difference between the control and Down syndrome with normal topography groups, “b” indicates a statistically significant difference between the control and Down syndrome with abnormal topography groups, “c” indicates a statistically significant difference between the Down syndrome with normal and abnormal topography groups. *** Comparison of the control and Down syndrome (total) groups (Student’s *t*-test (S), chi square test (C) or Mann-Whitney U test (M), *p* < 0.05 indicates statistical significance).

**Table 2:** Comparison of the corneal morphogeometric parameters between the control and Down syndrome groups.

Variables (Mean ± SD*)*	Control Group Normal Topography (*n* = 58)	Down Syndrome Normal Topography (*n* = 18)	Down Syndrome Abnormal Topography (*n* = 25)	Down Syndrome Group Total (*n* = 43)	3-Group Comparison * p	Pairwise Comparisons ** p	Down vs. Control Group Comparison *** p
**D_apexant_** (mm)	**0**	**0.003 ± 0.008**	**0.009 ± 0.010**	**0.007 ± 0.010**	**<0.0001**	**a, b (<0.0001 both)**	**<0.0001** (M)
**D_apexpost_** (mm)	0.071 ± 0.021	0.061 ± 0.038	0.086 ± 0.053	0.076 ± 0.048	0.058	-	0.406 (M)
**D_mctant_** (mm)	**0.841 ± 0.243**	1.268 ± 0.856	**1.585 ± 1.156**	**1.453 ± 1.042**	**0.007**	**b (0.009)**	**0.002 (M)**
**D_mctpost_** (mm)	**0.772 ± 0.236**	1.187 ± 0.842	**1.499 ± 1.121**	**1.368 ± 1.015**	**0.005**	**b (0.006)**	**0.002 (M)**
**A_ant_ (mm^2^)**	**43.09 ± 0.15**	**43.52 ± 0.17**	**43.41 ± 0.30**	**43.46 ± 0.25**	**<0.0001**	**a, b (<0.0001 both)**	**<0.0001** (S)
**A_post_** (mm^2^)	**44.24 ± 0.30**	**44.68 ± 0.37**	**44.54 ± 0.46**	**44.60 ± 0.42**	**<0.0001**	**a, b (<0.0001, 0.013)**	**<0.0001** (M)
**A_tot_** (mm^2^)	103.75 ± 1.22	103.55 ± 1.60	103.17 ± 1.46	103.33 ± 1.51	0.240	-	0.121 (S)
**A_apexant_** (mm^2^)	**0**	**4.00 ± 0.26**	**3.96 ± 0.32**	**3.98 ± 0.29**	**<0.0001**	**a, b (<0.0001 both)**	**<0.0001** (S)
**A_apexpost_** (mm^2^)	**4.27 ± 0.28**	**3.99 ± 0.26**	**3.95 ± 0.29**	**3.97 ± 0.27**	**<0.0001**	**a, b (0.003, <0.0001)**	**<0.0001** (S)
**A_mctpost_** (mm^2^)	**4.26 ± 0.27**	**3.97 ± 0.26**	**3.92 ± 0.29**	**3.94 ± 0.28**	**<0.0001**	**a, b (0.001, <0.0001)**	**<0.0001** (S)
**C_x_** (mm)	**0.041 ± 0.015**	**0.008 ± 0.038**	**−0.013 ± 0.052**	**−0.004 ± 0.048**	**<0.0001**	**a, b (0.01, <0.0001)**	**<0.0001** (S)
**C_y_** (mm)	**0.034 ± 0.025**	0.033 ± 0.041	0.011 ± 0.048	**0.020 ± 0.041**	0.056	-	**0.044** (S)
**C_z_** (mm)	**0.767 ± 0.023**	**0.787 ± 0.026**	0.782 ± 0.031	**0.784 ± 0.029**	**0.008**	**a (0.0160)**	**0.024** (M)

D_apexant_ and D_apexpost_: average distance from the Z axis to the highest point (apex) of the anterior and posterior corneal surfaces; D_mctant_ and D_mctpost_: average distance in the XY plane from the Z axis to the minimum thickness points of the anterior and posterior corneal surfaces; A_ant_ and A_post_: area of the anterior and posterior corneal surfaces; A_tot_: sum of anterior, posterior and perimetric corneal surface areas; A_apexant_ and A_apexpost_: sagittal plane apex area of the cornea within the sagittal plane passing through the Z axis and the highest point (apex) of the anterior and posterior corneal surfaces; A_mctpost_: sagittal plane area of the cornea within the sagittal plane passing through the Z axis and the minimum thickness points in the posterior corneal surfaces; (C_x_, C_y_, C_z_): centre of mass coordinates (X, Y, Z) of the solid; SD: standard deviation. *p* < 0.05 and bold values indicate statistical significance. * Comparison of the control group (non-Down subjects with normal topography), Down syndrome with normal topography and Down syndrome with abnormal topography subgroups (Kruskal–Wallis test, *p* < 0.05 indicates statistically significant difference). ** Pairwise comparisons among the three groups (*p* < 0.0166 indicates statistically significant difference after post-hoc corrections); “a” indicates a statistically significant difference between the control and Down syndrome with normal topography groups, “b” indicates a statistically significant difference between the control and Down syndrome with abnormal topography groups, “c” indicates a statistically significant difference between the Down syndrome with normal and abnormal topography groups. *** Comparison of the control and Down syndrome (total) groups (Student’s *t*-test (S) or Mann–Whitney U test (M), *p* < 0.05 indicates statistical significance).
